# Rosmarinic acid ameliorates acetaminophen-induced acute liver injury in mice via RACK1/TNF-α mediated antioxidant effect

**DOI:** 10.1080/13880209.2021.1974059

**Published:** 2021-09-13

**Authors:** Yang Yu, Yao Wu, Hao-zheng Yan, Zi-ru Xia, Wen Wen, Dan-yang Liu, Li-hong Wan

**Affiliations:** a Department of Neurosurgery, West China Hospital, Sichuan University, Chengdu, PR China; b Department of Pharmacology, West China School of Basic Medical Sciences & Forensic Medicine, Sichuan University, Chengdu, PR China; c NHC Key Laboratory of Chronobiology, Sichuan University, Chengdu, PR China; d West China School of Medicine, Sichuan University, Chengdu, PR China

**Keywords:** ALI, non-steroidal anti-inflammatory drug, antioxidative, receptor for activated C kinase 1, tumour necrosis factor-α

## Abstract

**Context:**

Rosmarinic acid (RA) dose-dependently ameliorates acetaminophen (APAP) induced hepatotoxicity in rats. However, whether RA hepatoprotective effect is by regulating RACK1 and its downstream signals is still unclear.

**Objective:**

This study explores the RA protective effect on APAP-induced ALI and its mechanism.

**Materials and methods:**

Sixty Kunming mice 6–8 weeks old were randomly separated into six groups (*n* = 10) and pre-treated with normal saline, ammonium glycyrrhetate (AG) or RA (10, 20 or 40 mg/kg i.p./day) for two consecutive weeks. Then, APAP (300 mg/kg, i.g.) was administrated to induce ALI, except for the control. Serum alanine/aspartate aminotransferases (ALT and AST), malondialdehyde (MDA), superoxide dismutase (SOD) and histopathology were used to authenticate RA effect. The liver RACK1 and TNF-α were measured by western blot.

**Results:**

Compared with the APAP group, different dosages RA significantly decreased ALT (52.09 ± 7.98, 55.13 ± 10.19, 65.08 ± 27.61 U/L, *p* < 0.05), AST (114.78 ± 19.87, 115.29 ± 31.91, 101.78 ± 21.85 U/L, *p* < 0.05), MDA (2.37 ± 0.87, 2.13 ± 0.87, 1.86 ± 0.39 nmol/mg, *p* < 0.01) and increased SOD (306.178 ± 90.80, 459.21 ± 58.54, 444.01 ± 78.09 U/mg, *p* < 0.05). With increasing doses of RA, RACK1 and TNF-α expression decreased. Moreover, the RACK1 and TNF-α levels were positively correlated with MDA (*r* = 0.8453 and *r* = 0.9391, *p* < 0.01).

**Discussion and conclusions:**

Our findings support RA as a hepatoprotective agent to improve APAP-induced ALI and the antioxidant effect mediated through RACK1/TNF-α pathway.

## Introduction

Acetaminophen (APAP) is considered as a relatively safe antipyretic analgesic at therapeutic doses. However, an overdose of APAP severely threatens human health due to drug-induced acute liver injury (ALI) (Lee 2017). Moreover, long-term administration of APAP has been found to induce liver fibrosis in mice (Bai et al. 2017). Mechanistically, the unbalance of oxidation and antioxidation is one of the leaders of APAP hepatotoxicity.

The receptor for activated C-kinase 1 (RACK1) is a member of the tryptophan-aspartate repeat (WD-repeat) family of proteins, which has been identified as a critical regulator in cell cycle, survival, adhesion and migration (Wang et al. 2011). Multiple lines of evidence underscore that as a downstream target gene of transforming growth factor β1 (TGF-β1), RACK1 is involved in modulating liver fibrosis progression via interacting with ADAM metallopeptidase domain 12 (ADAM12) (Bourd-Boittin et al. 2008; Jia et al. 2013) and oxidative stress (OS) response with carbonyl reductase 1 (CBR1) (Núñez et al. 2009; Zhou et al. 2016). Interestingly, previous publications demonstrated the potential positive relationship of RACK1 and tumour necrosis factor-α (TNF-α) production in androgen and oestrogen stimulated THP-1 cells (Corsini et al. 2016; Buoso et al. 2017, 2020). Specifically, in LPS-induced THP-1 cells, both transcriptional activity and protein expression of RACK1 were increased after nandrolone treatment, an androgen receptor (AR) agonist, which paralleled with increased TNF-α production (Buoso et al. 2017). Thus, RACK1/TNF-α seems to act as a molecular mechanism of OS in response to APAP-induced hepatocyte damage.

Antioxidants have been reported to alleviate APAP-induced liver fibrosis by attenuating liver OS injury (Yan et al. 2018). Although *N*-acetylcysteine (NAC), as the glutathione (GSH) precursor, is the only approved clinical drug to treat patients with APAP overdose, its narrow therapeutic time window limits the clinical usage (Fisher and Curry 2019). Therefore, there is a desperate need to find new therapeutic targets and agents to extend the therapeutic time frame. Rosmarinic acid (RA) is an ester of caffeic acid; 3,4-dihydroxyphenyllactic acid is first isolated from rosemary (*Rosmarinus officinalis* L. [Lamiaceae]). Accumulating evidence indicates that RA exerts a gamut of beneficial biological activities, such as antioxidant, anti-inflammatory, even antifibrotic effects (Domitrović et al. 2013). Recently, a series of studies suggest that RA possesses beneficial effects on various liver damages, including CCl_4_ induced acute liver toxicity (Domitrović et al. 2013), alcohol-induced hepatotoxicity (Hasanein and Seifi 2018) and bile-duct ligation induced extrahepatic cholestasis (Lin et al. 2017) owing to its antioxidative effect. RA has also been shown to restore impaired liver function due to partial hepatectomy (Lou et al. 2016) and protective effect on APAP-induced hepatotoxicity in male Wistar rats by inhibiting hepatic CYP2E1 activity and lipid peroxidation (Hasanein and Sharifi 2017). However, no study has been carried out concerning the protective effects of RA on RACK1/TNF-α associated OS response to our knowledge.

Based on the literature evidence, we designed our study to investigate whether RA possessed its hepatoprotective effect through suppressing OS response by regulating RACK1/TNF-α in APAP-induced mice.

## Materials and methods

### Animals and experimental groups

Sixty male Kunming mice, aged 6–8 weeks and weighing 20 ± 2 g, were obtained from the Animal Experiment Centre of West China Hospital, Sichuan University (Chengdu, China). The mice were acclimated for one week under standard conditions of ambient temperature (22 °C ± 1 °C), humidity (60%±10%) and with a 12 h light/dark cycle (lights on at 8:00 am). All experimental procedures were conducted in accordance with the Guidelines of the Experimental Research Institute of Sichuan University in agreement with the guidelines of the Canadian Council on Animal Care (permit no.: 2003-149). All the animal experiments were approved by the Ethical Committee for Laboratory Animals of West China School of Basic Medical Sciences & Forensic Medicine.

All mice were randomly divided into six groups (*n* = 10), including the control (Con), the APAP (Shanghai Johnson Pharmaceutical Co., Ltd., Shanghai, China), the ammonium glycyrrhetate (AG, 200 mg/kg), the low dose of RA (donated by Wenlong Deng, purity 99% by HPLC) (LRA, RA 10 mg/kg), the middle dose of RA (MRA, RA 20 mg/kg) and the high dose of RA (HRA, RA 40 mg/kg). All the materials under study are endotoxin free.

### APAP-induced acute hepatotoxicity mice model and treatment

**Figure 1.**
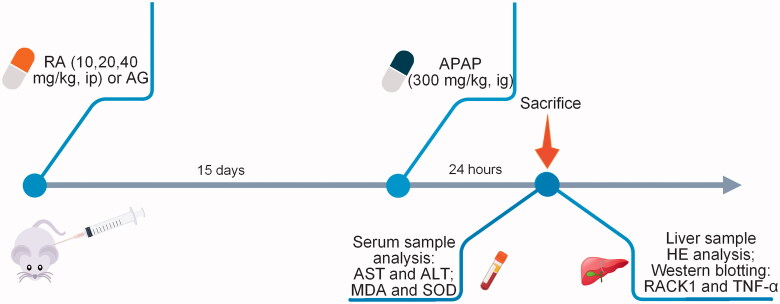
The schematic diagram of the treatment schedule.

Based on preliminary studies (Hasanein and Sharifi 2017), we dissolved RA (10, 20 or 40 mg/kg/d) and AG in sterilized normal saline and administered each via gavage to mice in the three RA groups and AG group for consecutive 15 days. The control and APAP groups received 2 mL sterilized normal saline via gavage. All mice were fasted overnight before APAP administration. On day 16, paracetamol suspension drops were administered by gavage (300 mg/kg) to induce APAP acute hepatotoxicity, according to previous publications (Dara et al. 2015). The control group accepted intragastric administration with sterilized normal saline. On day 17, all mice were euthanized with pentobarbital (50 mg/kg i.p. dissolved in sterilized NS) and decapitated. Serum samples were collected for analysis of alanine transaminase (ALT) and aspartate transaminase (AST). Liver tissues were harvested, the livers from five animals in each group were immediately fixed in 10% (v/v) neutral-buffered formalin for 24 h at 4 °C and embedded in paraffin for histological examination. Parts of mice livers in each group were rinsed in a cold phosphate buffer solution and divided into two parts. One part was temporarily stored at −80 °C for the following protein extraction and Western blot analysis. Another part was stored at 4 °C for the following biochemical analysis. The schematic diagram of the treatment schedule is shown in Figure 1.

### Serum and hepatic tissue biochemical analysis

Blood samples were collected from pentobarbital anaesthetized animals by cardiac puncture. About 300 µL serum was collected from whole blood after centrifugation at 4000 rpm for 10 min at 4 °C. As markers of hepatic function, the enzymatic activities of ALT and AST were determined by rate assay (C052 & C072, Changchun Huili Biotech, Changchun, China). The absorbance of the sample was detected at 340 nm.

Each liver tissue was ground with an appropriate amount of sterilized normal saline, then the grinding solution was centrifuged at 4000 rpm for 10 min at 4 °C. Then, the supernatant was collected for the activities determination of malondialdehyde (MDA) and superoxide dismutase (SOD) in the liver as markers of oxidation stress. MDA and SOD were measured using the TBA method and WST-1 method (Nanjing Jiancheng Institute of Biotechnology, Nanjing, China). The absorbance of sample was detected at 532 nm and 550 nm for MDA and SOD.

### Liver tissue histopathology

After fixation and embedding, five hepatic tissue samples in each group were sectioned at 4 μm thickness and stained with haematoxylin and eosin (HE) (Sigma, St. Louis, MO). An experienced pathologist evaluated all histological changes, including inflammation and hepatocellular damage in a blinded manner under light microscopy. The total score ranges from 0 to 10 (Hu 2012), which is based on a semi-quantitative analysis of the four definer criteria of non-alcoholic steatohepatitis: steatosis (0–2), cholestasis (0–1), apoptotic (0–2) and lobular inflammation (0–5).

### Molecular docking

Molecular docking was performed with Accelrys Discovery Studio 4.0 (DS, BIOVIA Software, Inc., San Diego, CA) to explore the possible binding between RA and RACK1. The crystal structure of RACK1 was obtained from the Protein Data Bank (PDB). The pre-processed receptor and ligand were then batch processed using MGLTooLs-1.5.6 to generate files in pdbqt format. Then, the process of molecular docking was performed with AutoDock Vina. Various binding modes were evaluated based on the docking energy, and the score was obtained. Reasonable docking results were selected according to the lowest score. Concretely, the docking result is feasible when binding energy is less than −1.2 kcal/mol.

### Protein preparation and Western blotting analysis

Total liver protein was extracted with RIPA Lysis Buffer (Beyotime Institute of Biotechnology, Shanghai, China). Protein concentrations were determined using the BCA protein assay kit (Beyotime Institute of Biotechnology, Shanghai, China).

Equal amounts of total protein were loaded onto 10% SDS-PAGE gels. Loaded proteins were separated at 100 V for 60 min and electrotransferred to PVDF membranes by the wet transfer method (200 mA, 60 min). The membranes were blocked in 5% fat-free milk at 4 °C overnight. Subsequently, the membranes were incubated with primary antibody at room temperature for 2 h, including anti-β-actin (ZSGB-BIO, Beijing, China) antibody (dilution 1:1000), anti-RACK1 (Abcam, Cambridge, MA) antibody (dilution 1:1000) and anti-TNF-α mAb (Abcam, Cambridge, MA) antibody (dilution 1:1000). After washing with TBST, the membranes were incubated with secondary goat anti-mouse IgG (ZB-2305, ZSGB-BIO, Beijing, China) antibody (dilution 1:1000) at room temperature for 1 h, and were conjugated with horseradish peroxidase. Then, proteins were detected by a chemiluminescence reagent (GE Healthcare, Chicago, IL). Protein band density was quantified using Bio-Rad Quantity One v4.62 (Hercules, CA). The relative protein levels were normalized to β-actin level.

### Statistics

Statistical analysis was performed by one-way analysis of variance (ANOVA) with Bonferroni’s correction. Spearman’s rank correlation coefficient was calculated for correlation analysis (GraphPad Prism version 5; GraphPad Software, La Jolla, CA). All values were expressed as mean ± SEM, and *p* < 0.05 was considered to be statistically significant.

## Results

### Rosmarinic acid alleviates APAP-induced acute liver injury in mice

**Figure 2.**
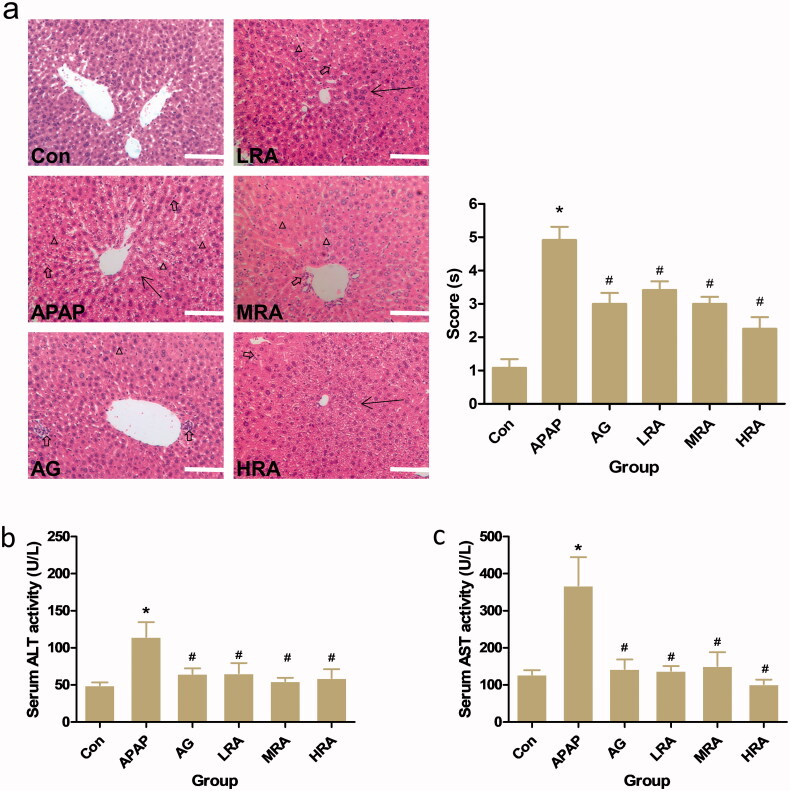
Protective effects of rosmarinic acid on APAP-induced acute liver injury in mice. (a) Histology. Histological examination of liver tissue was performed 24 h after 300 mg/kg APAP administration. Liver tissues were fixed, sectioned at 4 μm thickness, and stained with haematoxylin–eosin (HE). Representative HE (left panel) stained sections of the liver of mice in the control group (Con), APAP group, APAP + ammonium glycyrrhetate group (AG), APAP + RA (10 mg/kg) group (LRA), APAP + RA (20 mg/kg) group (MRA) and APAP + RA (40 mg/kg) group (HRA) (magnification ×200); Histological scores (right panel) were evaluated by an experienced pathologist in a blinded manner under light microscopy (*n* = 5). Triangles display vacuolization. The arrows represent the necrosis with the abnormal structure of hepatic lobules. The hollow arrows show the mononuclear cell infiltration. (b) Serum alanine aminotransferase (ALT) (*n* = 10). (c) Serum aspartate aminotransferase (AST) (*n* = 10). Values are presented as mean ± S.E.M. *p < 0.05 vs. control group. ^#^*p* < 0.01 vs. APAP group.

To confirm the potential hepatic protective effect of RA in mice, mice were pre-treated with RA (10, 20 or 40 mg/kg) and AG (200 mg/kg) for 15 days prior to APAP administration (300 mg/kg). The histopathologic analysis was performed on HE stained hepatic sections. As shown in Figure 2(a), APAP administration significantly induced severe hepatocellular injury compared with the control mice. Loss of hepatocyte architecture (arrows), vacuolization of hepatocytes (triangles), massive necrosis and mononuclear cell infiltration (hollow arrows) in the portal area can be observed. Conversely, treatment with RA or AG significantly ameliorated the hepatocellular injury around the portal area, with hepatic parenchyma without necrosis with centrilobular area preserved and poorly infiltrated cells.

Moreover, after an APAP overdose of 300 mg/kg administration, the degree of liver injury was assessed using serum ALT and AST, which were notably increased compared to controls (Figure 2(b,c)). Treatment with RA or AG could rescue mice from APAP-induced ALI, as revealed by decreased ALT and AST (Figure 2(b,c)).

### Rosmarinic acid represses APAP-induced oxidative stress in mice

**Figure 3.**
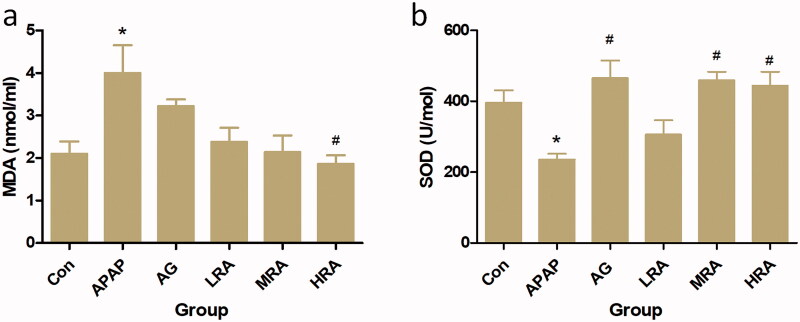
Protective effects of rosmarinic acid on oxidative stress parameters. (a) Serum malondialdehyde (MDA) content; (b) serum superoxide dismutase (SOD). Values are presented as mean ± S.E.M. *p < 0.05, vs. control group (*n* = 10). ^#^*p* < 0.01 vs. APAP group (*n* = 10).

Oxidative stress is closely related to liver injury. To investigate whether OS occurs in APAP-challenged mice, we examined the oxidation key markers' levels, including MDA and SOD in the serum. As shown in Figure 3(a,b), compared with the control group, the level of MDA in the APAP group was significantly increased (*p* = 0.0129), and the level of SOD in the APAP group was significantly reduced (*p* = 0.0486). However, pre-treatment with RA (40 mg/kg) could substantially reduce the serum MDA level and elevate the serum SOD level compared with the APAP treated group. All the results suggest that RA (40 mg/kg) had a marked antioxidant activity and could suppress APAP-induced oxidative injury.

### Rosmarinic acid potentially targets the RACK1/TNF-α signalling pathway to suppress oxidative injury

**Figure 4.**
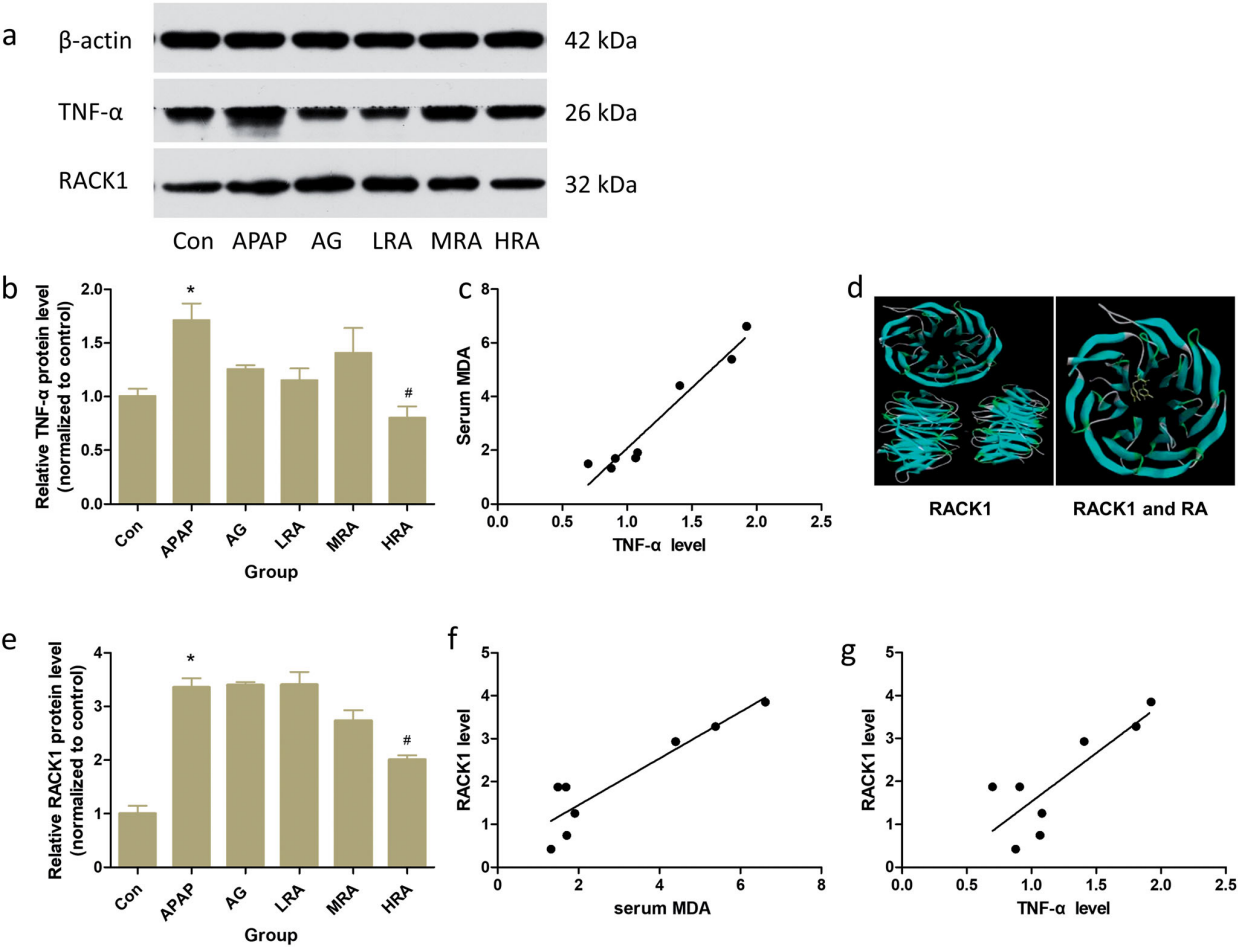
Effects of rosmarinic acid on RACK1/TNF-α signalling pathway. (a) The protein expression of TNF-α and RACK1 were analysed by western blotting. β-actin was used as an internal standard. (b) The relative level of TNF-α in liver. The results were expressed as the ratio of the investigated gene to β-actin. (c) Positive correlation of TNF-α protein level with serum MDA (*r* = 0.9391, *p* < 0.01). (d) Molecular docking result of RACK1 and RA. The crystal structure of RACK1 was blue and RA was yellow. The binding energy of RACK1 and RA was –7.4 kcal/mol. (e) The relative level of RACK1 in liver. The results were expressed as the ratio of the investigated gene to β-actin. (f) Positive correlation of RACK1 protein level with serum MDA (*r* = 0.8453, *p* < 0.01). (g) Positive correlation of RACK1 protein level with TNF-α protein level (*r* = 0.6778, *p* < 0.01). Values are presented as mean ± S.E.M. *p < 0.05 vs. control group (*n* = 8). ^#^*p* < 0.01 vs. APAP group (*n* = 8).

To elucidate the hypothesis that RA repressed oxidative injury was coupled to a decreased TNF-α level, we detect TNF-α expression through western blotting analysis. Also, its correlation with MDA was analysed. As we predicted, RA (40 mg/kg) treatment significantly decreased TNF-α expression level in APAP-challenged mice (Figure 4(a,b)). Spearman’s rank correlation coefficient was calculated to determine the relationship of protein and oxidative product. There was a significant positive correlation between TNF-α level and oxidative injury (MDA level) in mice (Figure 4(c)) (*r* = 0.9391, *p* < 0.01), with higher TNF-α protein expression predicting a higher degree of oxidative injury in mice.

According to the molecular docking result, RA could bind well with RACK1 with a binding affinity of −7.4 kcal/mol (Figure 4(d)). To further explore whether RA's inhibitory effect on APAP challenge was associated with the reduction in RACK1, the protein expression level of RACK1 in the liver was analysed with western blotting. Our data demonstrated that the protein expression level of RACK1 was significantly elevated in APAP-challenged mice compared with that in saline-challenged mice (*p* < 0.05, Figure 4(a,e)), which is significantly positively correlated with oxidative injury (serum MDA level) in mice (Figure 4(f)) (*r* = 0.8453, *p* < 0.01), with higher RACK1 protein expression predicting a higher degree of oxidative injury in mice. Moreover, there was also a significant positive correlation between RACK1 and TNF-α levels in mice (Figure 4(g)) (*r* = 0.6778, *p* < 0.01), with higher RACK1 protein expression predicting a higher level of TNF-α in mice. Similar to TNF-α, RA (40 mg/kg) treatment notably reversed this elevation (*p* < 0.05). Collectively, these results suggest that RA (40 mg/kg) may effectively limit the APAP-induced ALI by down-regulating OS related RACK1/TNF-α signalling pathway.

## Discussion

APAP overdose induced ALI is the main threat for its application due to APAP-induced OS response overwhelms hepatocytes defences (Jadeja et al. 2015). Briefly, overdosed APAP enhanced CYP2E1 activity leads to excessive *N*-acetyl-*p*-benzoquinoneimine (NAPQI) generated to cause depletion of GSH and induce OS to result in hepatocyte necrosis (McGill and Jaeschke 2013) even liver injury (Huo et al. 2017). Therefore, inhibition of OS might become a target for the intervention of APAP-induced ALI. Based on this point, NAC is the only FDA-approved antidote in treating patients with APAP overdose (Saito et al. 2010). Although NAC exerts the potent antioxidant property and has been documented to replenish GSH by providing cysteine to enhance GSH production (Aldini et al. 2018), adverse effects limit its wide application, including nausea, vomiting and rashes. Meanwhile, the therapeutic time window and debateable doses hindered its clinical application (Fisher and Curry 2019).

As a natural inducer of endogenous cellular antioxidant defence (Nadeem et al. 2019), RA provides a rational assumption for liver injury. The previous study has shown that RA significantly inhibited hepatic CYP2E1 activity and destruction of hepatic architecture at the dose of 50 and 100 mg/kg for rats, which was approximately equivalent to our doses for mice (Hasanein and Sharifi 2017). These similar results indicated the hepatoprotective effect of RA in APAP-induced ALI. Consistent with these findings in rats, our data also showed that RA could effectively inhibit vacuolization of hepatocytes, massive necrosis and mononuclear cell infiltration in the portal area, three characteristic components of hepatocellular injury in mice. In this process, the antioxidant effect of RA might relieve OS and modulate inflammation. Corresponding to the result, we found that the OS product and inflammatory factor decreased. Moreover, RA administration significantly inhibited serum ALT and AST. It has been also suggested that APAP induced MDA formation and decreases SOD activity aggravated oxidative damage promoted liver tissue damage (Wu et al. 2017). Excessive MDA as production of lipid peroxidation reflects potential OS (Hassan et al. 2005). With OS, extra ROS generates and triggers lipid peroxidation to cause cell destruction and death. SOD displayed the hepatic protection effect by the antioxidative action, which was a critical enzyme against ROS reaction (Leong et al. 2017). Therefore, RA exhibited hepatoprotective effects as an antioxidant to reduce serum liver enzymes (ALT and AST) and OS production (MDA and ROS) (Nadeem et al. 2019). Our results demonstrated that RA had significant antioxidant activity in APAP-induced ALI mice by reducing serum MDA level and elevating serum SOD level, especially at the dose of 40 mg/kg. Taken together, the results of this study suggested that RA possessed an antioxidant effect on APAP-induced hepatotoxicity.

Tumour necrosis factor-α, a well-known critical pro-inflammatory cytokine, is an inducer of OS through activation of reactive oxygen species (ROS) (Fischer and Maier 2015) and plays an important role in the development of APAP-induced hepatotoxicity (Tsai et al. 2018). Accumulating evidence indicates that TNF-α directly induced OS by the increased production of superoxide (O^2–^) (Pennathur and Heinecke 2007), NO (Luo et al. 2005) and ROS (Parker 2004) via its receptor TNF receptor 1 (TNFR1). Moreover, ROS amplified the TNF/ROS responses by activating the NF-κB pathway (Fischer and Maier 2015). Therefore, TNF-α was considered as the critical inducer in both OS and inflammation. Our results revealed a significant positive correlation between TNF-α protein level and serum MDA level in mice. Consistent with previous studies, APAP overdose induced markedly elevated hepatic expression of TNF-α protein (Osakabe et al. 2002), while this APAP-induced upregulation of TNF-α was efficiently inhibited by the treatment of RA at the dose of 40 mg/kg in mice.

**Figure 5.**
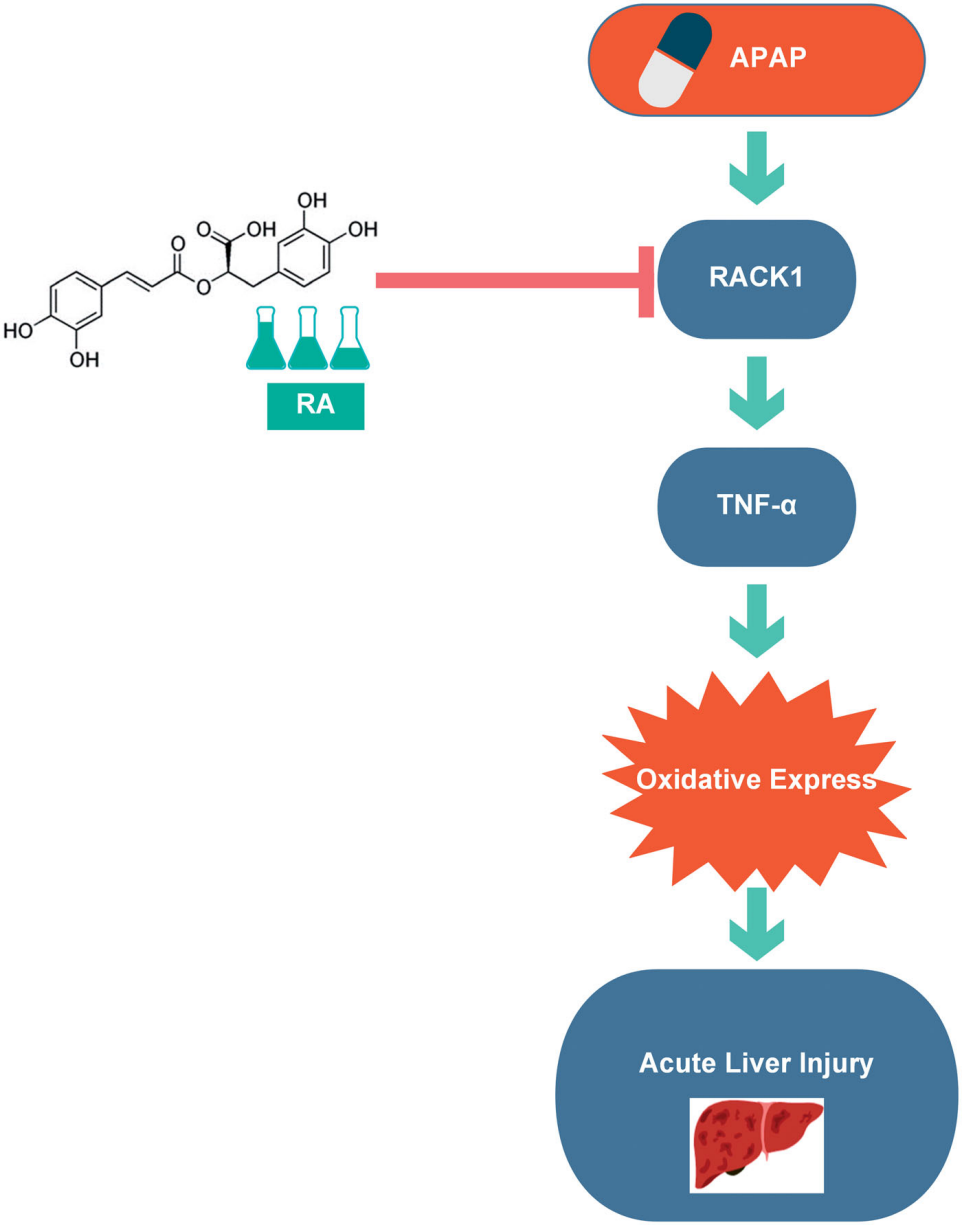
Graphical summary of the results. On exposure to APAP overdose, overexpression of RACK1 directly induced oxidative stress via TNF-α to prompt APAP hepatotoxicity. Rosmarinic acid suppresses oxidative stress by inhibiting the RACK1/TNF-α signalling pathway in APAP overdose induced acute liver injury.

RACK1, a regulator of TNF-α expression and release (Corsini et al. 2014; Zhang et al. 2018a, 2018b), is closely related to OS (Wang et al. 2013). It is worth noting that RACK1 displayed the potential protection effect from oxidative injury, including fission yeast (Núñez et al. 2009), *Penaeus monodon* (Saelee et al. 2011) and neuron (Ma et al. 2014). To better understand the precise role of RACK1 in the oxidative injury of APAP-induced hepatotoxicity, we measured the RACK1 protein level simultaneously and analysed the correlation between RACK1 and serum MDA. As we expected, similar to TNF-α expression, we also found a marked increase in hepatic expression of RACK1 protein. Simultaneously, RA (40 mg/kg) dramatically decreased RACK1 in the liver of APAP-induced mice. Additionally, the RACK1 protein level was positively correlated with the serum MDA. Also, we found that a significant positive correlation between RACK1 and TNF-α protein level in mice was consistent with Buoso’s and Corsini’s studies that both RACK1 transcriptional activity and protein level increased with accessorial TNF-α (Corsini et al. 2016; Buoso et al. 2020). However, in acute pancreatitis, both mRNA and protein levels of RACK1 were negatively correlated with TNF-α (Zhang et al. 2018a, 2018b), which was in stark contrast to our data and previous research (Corsini et al. 2016; Buoso et al. 2017, 2020). This difference might be caused by different disease models and the period of injury. It is essential to inhibit RACK1 as a control to determine the specific relationship of two proteins on ALI models in a future study. Moreover, the result of molecular docking could strengthen evidence of the potential function of RA. Thereby, these pieces of evidence have indicated that RA as the antioxidant could ameliorate APAP-induced hepatic injury and potentially interacted with RACK1/TNF-α, which might be used as a promising therapeutic way for protecting from APAP-induced acute liver failure (Figure 5).

## Conclusions

Our finding exhibited the protective effect of RA in APAP-induced ALI in mice, which could reduce serum liver enzymes and alleviate pathological change. Concretely, RA inhibits the RACK1/TNF-α signalling pathway and subsequently suppresses OS response, displaying as the alteration of MDA and SOD. Moreover, the level of TNF-α and RACK1 was positively correlated, indicating that RACK1 might function in the regulation of TNF-α in ALI. The present study may further offer basic information for the prevention and treatment of APAP overdose induced ALI.
